# Modeling antibiotic treatment in hospitals: A systematic approach shows benefits of combination therapy over cycling, mixing, and mono-drug therapies

**DOI:** 10.1371/journal.pcbi.1005745

**Published:** 2017-09-15

**Authors:** Burcu Tepekule, Hildegard Uecker, Isabel Derungs, Antoine Frenoy, Sebastian Bonhoeffer

**Affiliations:** Institute of Integrative Biology, ETH Zurich, Zurich, Switzerland; Oxford, UNITED KINGDOM

## Abstract

Multiple treatment strategies are available for empiric antibiotic therapy in hospitals, but neither clinical studies nor theoretical investigations have yielded a clear picture when which strategy is optimal and why. Extending earlier work of others and us, we present a mathematical model capturing treatment strategies using two drugs, i.e the multi-drug therapies referred to as cycling, mixing, and combination therapy, as well as monotherapy with either drug. We randomly sample a large parameter space to determine the conditions determining success or failure of these strategies. We find that combination therapy tends to outperform the other treatment strategies. By using linear discriminant analysis and particle swarm optimization, we find that the most important parameters determining success or failure of combination therapy relative to the other treatment strategies are the *de novo* rate of emergence of double resistance in patients infected with sensitive bacteria and the fitness costs associated with double resistance. The rate at which double resistance is imported into the hospital via patients admitted from the outside community has little influence, as all treatment strategies are affected equally. The parameter sets for which combination therapy fails tend to fall into areas with low biological plausibility as they are characterised by very high rates of *de novo* emergence of resistance to both drugs compared to a single drug, and the cost of double resistance is considerably smaller than the sum of the costs of single resistance.

## Author summary

For life-threatening infections, antibiotics need to be administered as soon as possible. Because it takes time to acquire data about the disease causing bacteria, the immediate treatment is often empiric. In particular, there are three treatment strategies discussed in the field of empiric treatment: cycling, mixing, and combination therapy. Despite a number of clinical and theoretical studies, it still remains unclear which treatment strategy best prevents the emergence of resistance and why. To address this controversy, we present a mathematical model capturing both mono- and multi-drug therapies. We sample and analyze a large parameter space to assess the effect of parameters on treatment success, and determine which treatment strategy is the best under which circumstances. Using methods such as linear discriminant analysis and particle swarm optimisation, we find that combination therapy outperforms the other strategies by a large margin for most of the biologically relevant parameter space. We also show that the rate of *de novo* emergence of double resistance and the costs of resistance mutations are the most important parameters determining whether combination therapy succeeds over the others.

## Introduction

Antibiotic resistance has become a global concern due to the increased emergence and spread of resistant bacteria [1–3]. Infections caused by resistant bacteria lead to higher mortality rates, higher treatment costs, and longer hospital stays [4, 5]. The main selection pressure that drives the emergence and spread of resistance is antibiotic use [6, 7]. Since it is not possible to abandon antibiotic usage completely, it is necessary to understand the dynamics of emergence and spread of resistance under drug pressure. Considering the decreasing rate at which new antibiotics are developed, it is critically important to use the available drugs in ways that delay the emergence and spread of resistance.

For life-threatening infections, antibiotics need to be administered as soon as possible to lower the mortality rate [8]. Clinical evidence shows that instant initiation of treatment is as important as chosing the appropriate treatment [9]. Since it takes time to acquire adequate data about the disease causing bacteria, immediate treatment is often empiric. Although current technology allows for faster and more accurate data collection, there are still large gaps in knowledge about the frequency of antimicrobial resistance worldwide [10]. Therefore empirical treatment still remains crucial, especially for resource-constrained facilities.

Multiple strategies are discussed in the field for such empiric treatment with the aim of reducing mortality and the risk of resistance within an open community, such as a hospital ward. In particular three strategies are discussed in more detail in the literature: (i) cycling, i.e. rotating between alternative antibiotics over a certain period; (ii) mixing, i.e. random assignment of different antibiotics to patients; (iii) combination therapy, i.e. combining multiple antibiotics to treat patients. Despite the large number of clinical studies, there is still a considerable debate regarding the potential benefits of these treatment strategies. Combination therapy is typically compared to monotherapy in the empiric management of infection [11]. While *in vitro* and animal studies show benefits of combination over mono drug therapy, clinical data are conflicting and less clear [12]. Some clinical studies suggest that combination therapy decreases the mortality rate compared to monotherapy [13–16], whereas others only conclude that it is non-inferior but provides no advantage [17–21]. A number of studies investigate the benefits of cycling [22–29], and some of them compare cycling to mixing [25, 26]. Some studies report benefits [22–24], whereas others find no benefits or even risks [27–29]. The studies are difficult to compare because of methodological differences, differences in pathogens, and differences in clinical endpoints. Moreover, a review of clinical trials of cycling comes to the conclusion that many studies have methodological shortcomings such that it remains unclear whether cycling has any benefit [30].

While clinical trials are ultimately indispensable, mathematical models can help to gain a better understanding of the epidemiological dynamics under the various treatment strategies [31]. Analysis and simulation of mathematical models have been widely used to quantify the population-wide effects of different treatment strategies, and many studies address the use of multiple drugs to manage antibiotic resistance [32–41]. However, these studies differ in many aspects, impeding the emergence of a conclusive picture. They differ in the assumptions they make about the processes relevant to the emergence and spread of resistance, they differ in the criteria by which they assess the performance of the treatment strategies, and most of them do not consider all multi-drug treatment strategies for comparison. Moreover, they usually only explore a limited parameter range, which in return limits the potential outcomes. Although according to our reading of the literature, many mathematical models show advantages for combination therapy, it is diffucult to understand which strategy is optimal under which circumstances, and why.

Here, we set up a model that contains the most essential processes of existing models and probe the performance of all three multi-drug therapies: cycling (CYC), mixing (MIX), and combination therapy (COMBO). We include mono-drug therapy as a reference, since there is no a priori reason to assume multi-drug therapies will perform better considering all consequences of treating a patient, such as selecting for double resistance. The model assumes that resistant cases in the hospital are either due to the admission of patients carrying resistant strains, the spread of resistance in the hospital, or the *de novo* emergence of resistance in treated patients. The *de novo* emergence of single and double resistance is modeled by two independent rates. The model can be considered as a combination of the models proposed by Bonhoeffer et al. 1997 [32] and Bergstrom et al. 2004 [33].

We systematically explore the parameter space to determine which treatment strategy performs the best under which circumstances. Using Linear Discriminant Analysis (LDA), we identify the key parameters for the success of each treatment strategy. We furthermore identify the parameter regions where COMBO is either the best or the worst strategy. We find that COMBO wins by a large margin in most of the realistic parameter space, but there are also parameter regions where it fails considerably.

## Methods

### Mathematical model

**Fig 1:**
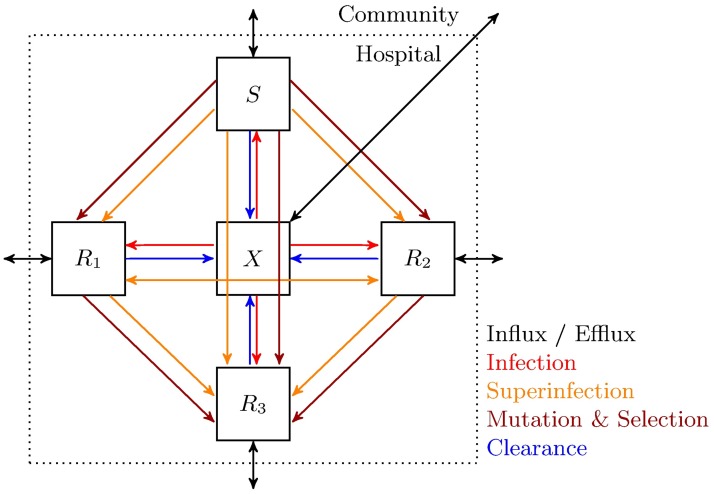
Schematic diagram of the dynamical model given by Eqs 1–5. The variables *X*, *S*, *R*_1_, *R*_2_, and *R*_3_ represent uninfected patients, and patients infected with a sensitive strain, a strain resistant to drug 1, a strain resistant to drug 2, and a strain resistant to both drugs, respectively.

Our mathematical model describes the transmission and spread of antibiotic resistance in an open system such as a hospital ward, in which patients are treated with two different antibiotics, denoted as drug 1 and drug 2. The model is a combination of two models described in [32] and [33], and depicted in Fig 1. Following a well established tradition in the field of antibiotic resistance modeling in hospitals [32, 33, 37], we do not explicitly represent the populations of bacteria within patients (no explicit within-host dynamics). Instead at each time point each patient belongs to one of 5 compartments, and our model consists in a set of differential equations describing how patients move from one compartment to another. The compartments *X*, *S*, *R*_1_, *R*_2_, and *R*_3_ represent the patients that are uninfected, infected with a sensitive strain, infected with a strain resistant to drug 1, infected with a strain resistant to drug 2, and infected with a strain resistant to both drugs, respectively.

In general, there are four processes that are taken into account to describe the flow of individuals between different compartments: infection, superinfection, *de novo* emergence of resistance, and clearance. Additionally, there are the process of influx and efflux of patients into and from all compartments, which means that patients can enter and leave the hospital ward independently of their current status. Patients are assumed to leave (and enter) the hospital with a constant rate of *μ*. Fractions of patients entering the hospital in the states *S*, *R*_1_, *R*_2_, *R*_3_, and *X* are denoted by *m*_0_, *m*_1_, *m*_2_, *m*_3_, and (1 − *m*_0_ − *m*_1_ − *m*_2_ − *m*_3_), respectively.

Uninfected patients get infected with a rate proportional to the transmission rate of infection *β* and the frequency of the infecting compartment, modulated by the fitness cost of resistance of the corresponding infectious strain. For the sake of simplicity, we assume that the patients that belong to the *X* compartment are not infectious, and the patients that only belong to *S*, *R*_1_, *R*_2_, and *R*_3_ compartments transmit the pathogen. Fitness costs of resistance to drug 1, drug 2, and both drugs are denoted by *c*_1_, *c*_2_, and *c*_3_, respectively. We do not explicitly model within-host dynamics, and the fitness cost of a resistance allele is modeled as a reduction of the infectivity of the resistant strain. Mathematically speaking, the term (1 − *c*_*n*_) is a multiplicative factor reducing the ability of the resistant *R*_*n*_ strain to propagate to other patients. Having a different parameter *c*_3_ for the cost of double resistance instead of additively or multiplicatively combining the costs *c*_1_ and *c*_2_ allows to represent epistatic interactions.

The acquisition of resistance during treatment occurs via *de novo* emergence or superinfection. In this context, gaining single resistance means either susceptible strains becoming resistant to one of the drugs, or single resistant strains becoming double resistant. Gaining double resistance refers to susceptible strains becoming double resistant. *De novo* emergence is assumed to occur only under drug pressure, with rates *ν* and *q* for single and double resistance, respectively. The rates *ν* and *q* thus include both the mutation and selection processes. In this model, superinfection is defined as infection of an already infected patient with another strain, and depends on the relative rate of superinfection *σ*. We assume that a patient is infected by only one strain at a given time and do not consider the co-existence of multiple strains, which implies that superinfection results in the replacement of the former strain by the latter. Superinfection is assumed to occur under treatment, because the fitness difference of resistant and sensitive strains will be larger under drug pressure and superinfection will not play as important a role in the absence as in the presence of treatment. Moreover, since superinfection by a sensitive strain will be very unlikely under drug pressure, it is assumed that there is no flow to *S* compartment from *R*_1_, *R*_2_, and *R*_3_ compartments. Likewise, superinfection of *R*_3_ by *R*_1_ or *R*_2_ is assumed to be absent. Reversal of resistance is considered negligible; otherwise, superinfection by the sensitive strain would be possible in the absence of treatment.

Clearance includes both spontaneous recovery due to the patient’s immune system and due to treatment. Spontaneous recovery occurs a rate *γ*, and recovery due to appropriate treatment occurs at a rate of *τ*. Having a single recovery rate due to treatment stems from an underlying assumption about drug action: we assume that any treatment that works leads to recovery with the same rate independent of the treatment type, meaning that combining two drugs does not lead to faster clearance. This is a conservative assumption giving no direct treatment benefit to combination therapy, as in clinical trials it has often been found that monotherapy is noninferior (see Introduction).

The fractions of infected patients receiving drug 1, drug 2, or both drugs according to the current treatment protocol are denoted by *f*_1_, *f*_2_, and *f*_3_, respectively, and the total fraction of infected patients receiving drugs is *f*_*tot*_ = *f*_1_ + *f*_2_ + *f*_3_. Manipulating *f*_1_, *f*_2_, and *f*_3_ determines the current treatment strategy, which may be monotherapy with drug 1 (MONO-1) or drug 2 (MONO-2), mixing (MIX), cycling (CYC), or combination therapy (COMBO). Following Bergstrom et al. 2004, we also consider “erroneous” treatment reflecting that not all patients may receive treatment according to the chosen treatment strategy. This erroneous rate of treatment is given by *a*_1_, *a*_2_, and *a*_3_, describing the rate of erroneous treatment with drug 1, drug 2, or both in combination. For simplicity, we assume that *a*_1_ = *a*_2_ = *a*_3_ = *a*.

The schematic given in Fig 1 can be translated into a system of ordinary differential equations, where each arrow, i.e., each process, is associated with a rate. This system is given by Eqs (1)–(5), including the rates of processes as model parameters, and describes the rate of change of compartments over time. Model parameters are given in Table 1 with their corresponding description, unit, and process.

$$\begin{array}{ll} dS/dt= & m_{0}\mu -\mu S-\nu \left(f_{1}+a_{1}\right)S-\nu \left(f_{2}+a_{2}\right)S-q\left(f_{3}+a_{3}\right)S-\left(\tau +\gamma \right)S+\beta SX-\left(f_{1}+a_{1}\right)\sigma \beta \left(1-c_{1}\right)R_{1}S\\ & -\left(f_{2}+a_{2}\right)\sigma \beta \left(1-c_{2}\right)R_{2}S-\left(f_{1}+f_{2}+f_{3}+a_{1}+a_{2}+a_{3}\right)\sigma \beta \left(1-c_{3}\right)R_{3}S, \end{array}$$ (1)

$$\begin{array}{ll} dR_{1}/dt= & m_{1}\mu -\mu R_{1}+\nu \left(f_{1}+a_{1}\right)S-\nu \left(f_{2}+f_{3}+a_{2}+a_{3}\right)R_{1}-\left(\tau \left(f_{2}+f_{3}+a_{2}+a_{3}\right)+\gamma \right)R_{1}+\beta \left(1-c_{1}\right)R_{1}X\\ & +\left(f_{1}+a_{1}\right)\sigma \beta \left(1-c_{1}\right)R_{1}S+\left(f_{1}+a_{1}\right)\sigma \beta \left(1-c_{1}\right)R_{1}R_{2}-\left(f_{2}+a_{2}\right)\sigma \beta \left(1-c_{2}\right)R_{1}R_{2}\\ & -\left(f_{2}+a_{2}+f_{3}+a_{3}\right)\sigma \beta \left(1-c_{3}\right)R_{1}R_{3}, \end{array}$$ (2)

$$\begin{array}{ll} dR_{2}/dt= & m_{2}\mu -\mu R_{2}+\nu \left(f_{2}+a_{2}\right)S-\nu \left(f_{1}+f_{3}+a_{1}+a_{3}\right)R_{2}-\left(\tau \left(f_{1}+f_{3}+a_{1}+a_{3}\right)+\gamma \right)R_{2}+\beta \left(1-c_{2}\right)R_{2}X\\ & +\left(f_{2}+a_{2}\right)\sigma \beta \left(1-c_{2}\right)R_{2}S-\left(f_{1}+a_{1}\right)\sigma \beta \left(1-c_{1}\right)R_{1}R_{2}+\left(f_{2}+a_{2}\right)\sigma \beta \left(1-c_{2}\right)R_{1}R_{2}\\ & -\left(f_{1}+a_{1}+f_{3}+a_{3}\right)\sigma \beta \left(1-c_{3}\right)R_{2}R_{3}, \end{array}$$ (3)

$$\begin{array}{ll} dR_{3}/dt= & m_{3}\mu -\mu R_{3}+q(f_{3}+a_{3})S+\nu (f_{2}+f_{3}+a_{2}+a_{3})R_{1}+\nu (f_{1}+f_{3}+a_{1}+a_{3})R_{2}-\gamma R_{3}+\beta (1-c_{3})R_{3}X\\ & +\sigma \beta (1-c_{3})R_{3}S+(f_{2}+a_{2}+f_{3}+a_{3})\sigma \beta (1-c_{3})R_{1}R_{3}+(f_{1}+a_{1}+f_{3}+a_{3})\sigma \beta (1-c_{3})R_{2}R_{3}, \end{array}$$ (4)

$$\begin{array}{ll} dX/dt= & (1-m_{0}-m_{1}-m_{2}-m_{3})\mu -\mu X+(\tau +\gamma )S+(\tau (f_{2}+f_{3}+a_{2}+a_{3})+\gamma )R_{1}\\ & +(\tau (f_{1}+f_{3}+a_{1}+a_{3})+\gamma )R_{2}+\gamma R_{3}-\beta SX-\beta (1-c_{1})R_{1}X-\beta (1-c_{2})R_{2}X-\beta (1-c_{3})R_{3}X. \end{array}$$ (5)

**Table 1:** Model parameters with their corresponding description, unit, and process.

Parameter	Description	Unit	Process
*m*_0_, *m*_1_, *m*_2_, *m*_3_	Influx fractions (Admission of patients to the hospital ward in the states of *S*, *R*_1_, *R*_2_, and *R*_3_)	−	Influx / Efflux
*μ*	Turnover rate (Loss of patients due to discharge or death, compensated by admission of new patients)	day^−1^	
*β*	Transmission rate	day^−1^	Infection / Superinfection
*c*_1_, *c*_2_, *c*_3_	Fitness costs of resistance to drug 1, drug 2, and both drugs	−	
*σ*	Relative rate of superinfection	−	
*ν*	Rate of *de novo* emergence of single resistance due to drug treatment	day^−1^	Mutation & Selection
*q*	Rate of *de novo* emergence of double resistance due to drug treatment	day^−1^	
*γ*	Spontaneous recovery rate	day^−1^	Clearance
*τ*	Rate of recovery due to appropriate treatment	day^−1^	
*f*_*tot*_	Fraction of infected patients receiving drugs according to the current treatment protocol (*f*_*tot*_ = 1 − *a*_1_ − *a*_2_ − *a*_3_)	−	
*f*_1_, *f*_2_, *f*_3_	Fraction of infected patients receiving drug 1, 2, or both	−	
*a*_1_, *a*_2_, *a*_3_	Fraction of infected patients receiving drug 1, 2, or both, erroneously	−	

The different treatment strategies are simulated by manipulating the parameters *f*_1_, *f*_2_, and *f*_3_. MIX implies the random assignment of drugs 1 and 2 to the half of the treated patients, i.e., *f*_1_ = *f*_2_ = *f*_*tot*_/2, and *f*_3_ = 0. CYC implies that at any given time the same drug type is used in the whole population, but switches between both drugs with a certain cycling period, which is kept constant (30 days) for all simulations. This can be simulated by switching between *f*_1_ = *f*_*tot*_ and *f*_2_ = *f*_*tot*_ with a constant frequency. In order to avoid any bias, the initial drug type is chosen randomly. MONO-1 and MONO-2 assign the same drug type for the whole population, and are simulated by either setting *f*_1_ = *f*_*tot*_ or *f*_2_ = *f*_*tot*_ for the entire duration of treatment. COMBO assigns both drugs at the same time for the whole population, i.e., *f*_3_ = *f*_*tot*_ and *f*_1_ = *f*_2_ = 0.

Since the Eqs (1)–(5) cannot be solved analytically, we simulate the system numerically. Simulations are carried out as follows. For a given parameter set Φ, the system (given by Eqs (1)–(5)) is numerically approximated via the Fourth Order Runge-Kutta Method (RK−4) [42] with a step size of 0.01 days, in the absence of treatment. We let the system to reach its steady state before the application of any treatment, and the steady state values are then used as the initial conditions of the hospital ward. These initial conditions are in equilibrium with the community since we take the pre-existence of resistance prior to treatment into account through non-zero influx rates of resistance (*m*_1_, *m*_2_, and *m*_3_). After the system has reached its steady state, treatment starts, and we define the time onset of therapy as time 0. Simulations then continue in parallel for each of the five different treatment strategies, MIX, CYC, COMBO, MONO-1, and MONO-2, for the whole course of simulation duration. Afterwards, an efficacy score M(Ω|Φ) conditioned on the parameter set Φ is calculated for each treatment type, defined as

$$\begin{array}{cc} M(\Omega |\Phi ) & =\int_{0}^{T}\left[X(\upsilon |\Phi )-X(0|\Phi )\right]\;d\upsilon , \end{array}$$ (6)

where Ω denotes the type of treatment, and *T* denotes the time point where the simulation ends. We choose *T* = 360 days for all simulations throughout this article. A finite time frame is chosen in order to see the effects of all the parameters, including those whose action is only visible before a new steady state is established.

**Fig 2:**
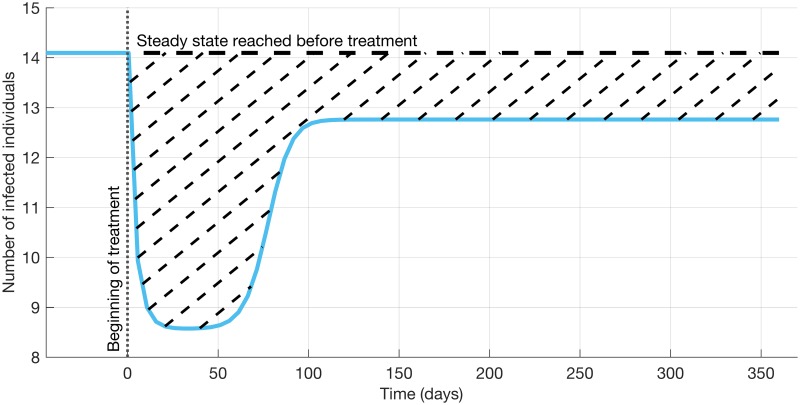
Illustration of the efficacy score. The score is calculated by integrating over the difference in the number of infecteds in the absence and presence of treatment, and the area representing the efficacy score is marked by the dashed lines.

The efficacy score quantifies the effect of a given treatment strategy on the sum of all infected patient classes compared to their number in the absence of treatment. Treatment strategies are compared based on their efficacy scores, and the strategy with the highest score is considered as the *winning strategy* for a given parameter set. An example graph of the time behaviour of the number of infected patients is given in Fig 2, where the efficacy score is illustrated with the dashed area.

### Classification and the optimization problem

We use two different approaches to explore the parameter space. Both approaches aim at identifying the regions where one strategy outperforms over the others. The first is based on random sampling (RS) of the parameter space. In order to separate parameter regions favoring one strategy over the others, Linear Discriminant Analysis (LDA) is applied on the parameter sets. The second approach directly searches for parameter regions where combination therapy performs significantly better or worse than the other treatment strategies. Due to the high dimensionality of the system, we employ the Particle Swarm Optimization (PSO) method to this optimization problem. For comparison, the identified regions are also projected on the same LDA space. Both approaches will be discussed in detail in the following sections.

#### Random sampling (RS)

RS involves the random sampling of a large number of parameter sets from the parameter space. Notice that, although the model has 21 parameters, there are only 15 free parameters due to the dependence of the parameters on each other and on the treatment strategy. As a result, each sampled parameter set is a vector of length 15, such that Φ = {*m*_0_, *m*_1_, *m*_2_, *m*_3_, *μ*, *β*, *c*_1_, *c*_2_, *c*_3_, *σ*, *ν*, *q*, *γ*, *τ*, *a*}.

All parameters are sampled within predefined parameter boundaries using either a logarithmic or a linear sampling scheme. Very broad parameter boundaries are chosen such that they are representative of the values used in the mathematical literature, and given in Table 2 with their corresponding sampling scheme.

**Table 2:** Model parameters given with their sampling range and sampling schemes.

Parameter	Range	Sampling
*m*_0_	[0.07, 0.9]	logarithmic
*m*_1_	[10^−3^, 10^−1^]	logarithmic
*m*_2_	[10^−3^, 10^−1^]	logarithmic
*m*_3_	[10^−6^, 10^−1^]	logarithmic
*μ*	[3 × 10^−3^, 5 × 10^−1^]	logarithmic
*β*	[0.2, 1]	linear
*c*_1_, *c*_2_, *c*_3_	[0, 0.2]	linear
*σ*	[0, 0.5]	linear
*ν*	[10^−10^, 10^−1^]	logarithmic
*q*	[10^−10^, 10^−1^]	logarithmic
*γ*	[0, 0.2]	linear
*τ*	[0.1, 1]	linear
*a*	[0, 0.2]	linear

#### Linear discriminant analysis (LDA)

LDA is a supervised method for dimensionality reduction for classification problems [43]. Intuitively, LDA performs a rotation of the high dimensional cloud of points representing the parameter sets in a manner such that when projected to a space of lower dimensionality, the classes are maximally separated. Projection onto the first two such dimensions thus maximally separates the parameter sets that belong to different classes of treatment strategies in a 2-D space.

Here, each treatment strategy is considered as a different class for the LDA method. Parameter sets are normalized and labeled according to which strategy performs best. Afterwards, the projection matrix **w** is calculated such that upon projection, the centroids of different classes are maximally separated, and points in every class have the smallest scattering possible. Here we project the parameter sets onto the 2-D space spanned by the first two axes of LDA. The Cartesian coordinates [*x*, *y*] of each projected parameter set Φ* are calculated as

$$\begin{array}{ccc} \underset{\Phi_{2\times 1}^{*}}{\underset{︸}{\left[\begin{array}{c} x\\ y \end{array}\right]}}= & \underset{{\text{W}}_{2}{}_{\times 15}}{\underset{︸}{\left[\begin{array}{llll} w_{1,1} & w_{1,2} & \cdots & w_{1,15}\\ w_{2,1} & w_{2,2} & \cdots & w_{2,15} \end{array}\right]}}\times & \underset{\Phi_{15\times 1}}{\underset{︸}{\left[\begin{array}{c} m_{0}\\ m_{1}\\ \vdots \\ a \end{array}\right]}} \end{array}.$$ (7)

Eq (7) can also be interpreted as [*x y*]^⊤^ being the linear sum of the parameter vectors {[*w*_1,1_ *w*_2,1_]^⊤^, [*w*_1,2_ *w*_2,2_]^⊤^, …, [*w*_1,15_ *w*_2,15_]^⊤^} weighted by the parameters {*m*_0_, *m*_1_, …, *a*}. As a result, every parameter set can be projected on a 2-D space as the weighted sum of the parameter vectors. The magnitude and the direction of a parameter vector indicates how far and in which direction a point will move relative to its original position if that particular parameter changes by a certain amount. This means that the significance of each parameter in class separation depends on both the position and the magnitude of its corresponding vector. A vector with a large magnitude located purely on the *x*-axis is significant for separating the classes horizontally, whereas it is insignificant for the class separation on the vertical axis.

LDA performs optimally when the parameters are independent and normally distributed, and assumes that a linear combination of parameters separates the classes. Although we sample the model parameters independently, they are not normally distributed, and there is a nonlinear relationship between the frequency of the compartments and the model parameters. However, it is known that LDA frequently performs well even when the assumptions of independence and normality are violated [44]. Therefore, LDA might not be the optimal method for our problem, but it still gives a good idea about which parameter regions favor which strategies, and which parameters matter the most for class separation.

#### Particle swarm optimization (PSO)

PSO is a population-based optimization method inspired by the collective behaviour of animal populations [45]. It shares many similarities with evolutionary computation techniques such as genetic algorithms [46]. The optimization process starts with an initial “population” of solutions called *particles* located randomly on the solution space. Each solution is assigned a fitness value, which the optimization algorithm tries to maximize. All particles search for the optimal solution by communicating and sharing their local solutions and fitness values at each iteration. Every particle adjusts its velocity on the solution space depending on the best solution in their own history (*P*_*best*_), and also the best solution obtained so far by any particle in the population (*G*_*best*_). *G*_*best*_ is updated if the best solution among all particles at the current iteration is better than the best solution from the previous iterations. Given enough iterations, all particles are expected to converge to the same solution on the solution space. Pseudocode of this algorithm is given below, where ⊗ denotes element-by-element vector multiplication. Variables *z*_1_ and *z*_2_ are the tuning parameters of the algorithm, that determine the strength of attraction of the particles to *P*_*best*_ and *G*_*best*_. Randomness, which is required for good solution space exploration, is introduced via the random vectors *r*_1_ and *r*_2_. Each of the elements in *r*_1_ and *r*_2_ is sampled uniformly between 0 and 1 [47]. Note that the stochasticity introduced by *r*_1_ and *r*_2_ may also induce stochasticity in the results, which means different realizations of the algorithm may lead to different optimal solutions.

**Algorithm 1** Particle swarm optimization (PSO) algorithm

1: **for** each particle *p* **do**

2:  Initialize particle

3: **end for**

4: **for** number of iterations **do**

5:  **for** each particle *p* **do**

6:   Compute the fitness value

7:   If the fitness value is better than the best fitness value in history, set current solution as the new *P*_*best*_.

8:  **end for**

  Choose the particle with the best fitness value of all the particles as the global best particle (*G*_*best*_)

9:  **for** each particle *p* **do**

10:   Calculate particle velocity according to *v* ← *v* + *z*_1_ ⊗ *r*_1_ ⊗ (*P*_*best*_ − *p*) + *z*_2_ ⊗ *r*_2_ ⊗ (*G*_*best*_ − *p*)

11:   Update particle position according equation *p* ← *p* + *v*

12:  **end for**

13: **end for**

In the context of our problem, each particle is a parameter set, and the fitness value is calculated based on the efficacy score of all strategies. In order to identify the parameter regions where combination therapy significantly fails, we define the fitness value for a given particle *p* as

$$J_{\text{worst}}\left(p\right)=\min\{M(\text{mixing}|p),M(\text{cycling}|p),M(\text{mono}\;\text{drug}\;1|p),M(\text{mono}\;\text{drug}\;\text{2}|p)\}-M(\text{combination}|p).$$ (8)

By maximizing this difference, the algorithm finds parameter sets where combination therapy fails by a maximal margin. Note that it does not matter which strategy is the best.

Similarly, parameter regions where combination therapy wins by a maximal margin over the other strategies can be found by defining the fitness value as

$$J_{\text{best}}\left(p\right)=M(\text{combination}|p)-\max\{M(\text{mixing}|p),M(\text{cycling}|p),M(\text{mono}\;\text{drug}\;1|p),M(\text{mono}\;\text{drug}\;\text{2}|p)\}.$$ (9)

## Results

**Fig 3:**
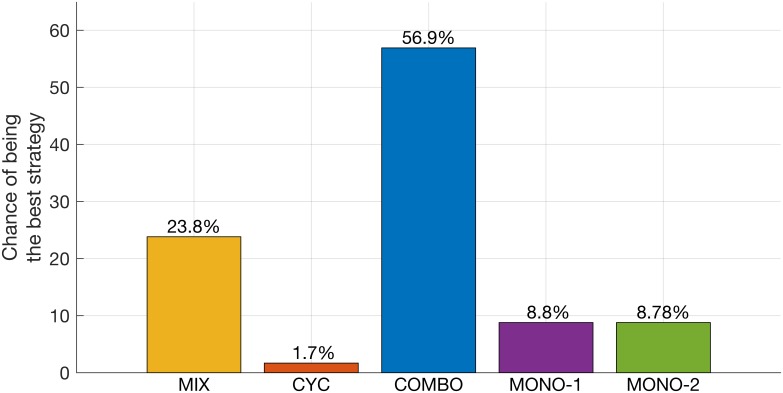
Chance of being the best strategy according to the random sampling results. 500,000 randomly sampled parameter sets are used. For each parameter set, efficacy scores are calculated for the five treatment strategies, and the winning strategy for that particular parameter set is determined. Based on these results, the probability of being the best strategy for each treatment is calculated.

In our analysis, we first examine the results of the random sampling (RS) algorithm. 500,000 parameter sets are sampled randomly according to the parameter boundaries and sampling schemes given in Table 2. For each parameter set, efficacy scores are calculated for the five treatment strategies, and the winning strategy for that particular parameter set is determined. Fig 3 shows the probability that a given strategy is the winning strategy based on all RS results. For random sampling of the entire parameter space, COMBO wins in 57% of the cases, which is substantially higher compared to the other strategies. In the parameter region where it is beneficial to treat a patient with more than one drug, COMBO tends to outperform CYC and MIX by being the best strategy 70% of the time. Note, however, that the overall chance of being the best strategy depends both on the choice of the parameter ranges and the sampling scheme. More statistical information on the distribution of efficacy scores and correlation between them is provided in S1 Table and S1 Fig.

It is the natural starting point to assume that the hospital ward is at equilibrium with the community before the application of any treatment. As mentioned in the Methods section, treatment-free steady state values for the fraction of different patient groups are used as the initial conditions when simulating the treatment strategies. Histograms of the resulting initial conditions are provided in the supplementary material (S5 Fig). However, to see wether using the equilibrium values as the initial conditions has an impact on the relative performances of the treatment strategies, we also ran simulations starting from randomly drawn initial conditions. A similar analysis for the chance of being the best strategy for randomly drawn initial conditions is provided in the supplementary material (S6 Fig), showing that for random initial conditions COMBO is even more often the best strategy.

**Fig 4:**
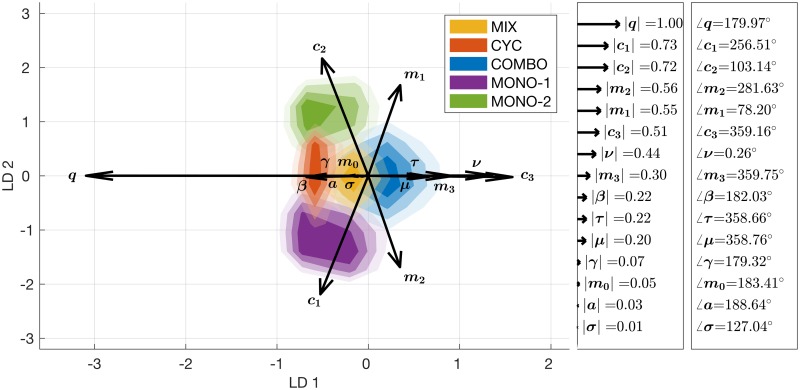
LDA of random sampling results. LDA classifying parameter sets according to the strategy with the highest efficacy score. Shaded areas represent the density of the parameter sets colored according to which strategy wins. Each treatment strategy is represented by a different color, and the opacity of each color is proportional to the number of parameter sets that fall into that corresponding region. 500,000 randomly sampled parameter sets are used. LD1 and LD2 are the two principal axes of the LDA. The parameter vectors are given with their relative magnitudes and counterclockwise angles. Parameter vectors are amplified by a factor of 3 in order to make them better visible. The most important parameters in terms of class separation are *q* (rate of *de novo* emergence of double resistance), *c*_1_, *c*_2_, and *c*_3_ (fitness costs of resistance to drug 1, drug 2, and both drugs).

To determine the parameter regions where particular strategies tend to win, the winning strategy for each particular parameter set is used as its class for linear discriminant analysis (LDA). These results are projected on a 2-D space using the projection matrix **w** = [LD1 LD2], and 2-D density plots of the projected samples are calculated for each class. These density plots are given in Fig 4, where the parameter vectors are amplified by by a factor of 3 in order to make them better visible. Counterclockwise angles and the relative magnitudes of the parameter vectors are also included.

As discussed in the Methods section, whether a parameter is significant for class separation or not depends both on the magnitude and the angle of its corresponding vector. For instance, as seen in Fig 4, *q* and *c*_3_ have the biggest influence on the separation of COMBO versus MIX and CYC, since the vectors of both parameters lie almost parallel to the horizontal axis. Although both *c*_1_ and *c*_2_ have similar magnitudes, the fact that they are aligned on the vertical rather than the horizontal axis indicates that they are more important for the separation of MONO-1 versus MONO-2. Overall, Fig 4 shows that *q* and *c*_3_ are the most important parameters separating the multi-drug therapies, whereas *c*_1_ and *c*_2_ are the most important parameters separating the mono-drug therapies. LDA of random sampling results only for multi-drug therapies is also provided in the supplementary material (S7 Fig).

**Fig 5:**
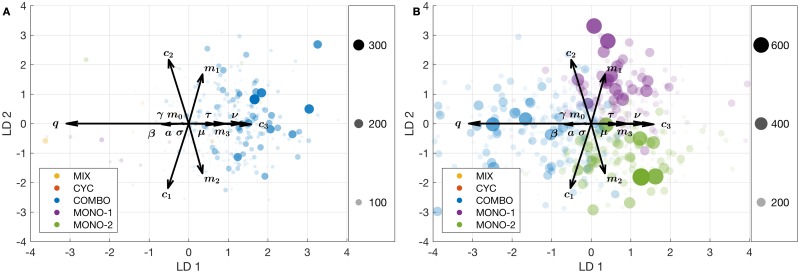
Marginal benefit comparisons for random sampling results. Results for the marginal benefit comparisons where 2000 samples are used. Disks are colored according to the winning strategy, and both the disk size and the transparency are proportional to the efficacy score difference between the best (worst) and the second best (second worst) strategy. Transparency values are normalized for each panel separately. Three examples of disks are provided with the values they represent on the right legend of each panel. LD1 and LD2 are the two principal axes of the LDA, and the most important parameters in terms of class separation are *q* (Rate of *de novo* emergence of double resistance), *c*_1_, *c*_2_, and *c*_3_ (Fitness costs of resistance to drug 1, drug 2, and both drugs). **(A)** Comparison of the best vs. the second best strategy. Disks are colored according to the best strategy. **(B)** Comparison of the worst vs. the second worst strategy. Disks are colored according to the worst strategy.

We will investigate the role of these parameters in more detail below. But before doing so, we first follow up another question, namely by which margin a strategy wins or loses compared to the second best and second worst, respectively. These comparisons show how right or wrong one can be by picking the best or the worst strategy over the next alternative. The results are shown in Fig 5, where a random subsample of size 2000 of the RS results are used. Disks are colored according to the winning strategy, and both the disk size and the transparency are proportional to the efficacy score difference between the best (worst) and the second best (worst) strategy. Fig 5A shows that the disks where COMBO wins (on the right hand side) are much bigger than the disks where other therapies win (on the left hand side), meaning that when combination therapy is the winning strategy, it wins by a large margin, whereas the other strategies when best are only negligibly better than the second best. In contrast, Fig 5B shows that no matter which therapy is the worst, the margin by which they fail compared to the second worst strategy is similar for all strategies. This implies that in certain parameter regions, COMBO can fail by a substantial margin. Whether this parameter region is biologically relevant or not is discussed further below. Marginal benefit comparisons between COMBO and MIX, COMBO and CYC, and MIX and CYC are provided in S2 Fig. In conclusion, the LDA results presented in Figs 4 and 5 show that the important parameters for determining the winning strategy are *q*, *c*_1_, *c*_2_, and *c*_3_.

**Fig 6:**
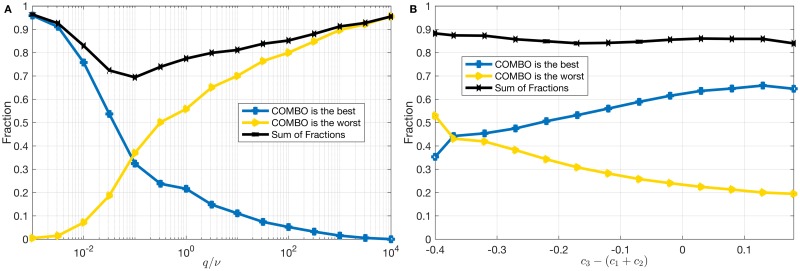
Analysis of the important parameters. Analysis of the role of *de novo* emergence of resistance for RS results. **(A)** Semi logarithmic plot of fraction of combination therapy being the best or the worst within all strategies vs. *q*/*ν*, (*q*: rate of *de novo* emergence of double resistance, *ν*: rate of *denovo* emergence of single resistance) for 500,000 RS results. **(B)** Plot of fraction of combination therapy being the best or the worst within all strategies vs. *c*_3_ − (*c*_1_ + *c*_2_) (*c*_1_,*c*_2_,*c*_3_: fitness costs of resistance to drug 1, drug 2, and both drugs), for 500,000 RS results.

To assess the role of *de novo* emergence of resistance on the efficacy of COMBO relative to other strategies, we show the influence of *q* (rate of *de novo* emergence of double resistance) relative to the influence of *ν* (rate of *de novo* emergence of single resistance), and the fraction of parameter sets for which combination therapy is the best or the worst strategy for a given *q*/*ν* value. As can be seen in Fig 6A, COMBO is often the best and rarely the worst strategy as long as *q* ≪ *ν*. It fails increasingly as *q*/*ν* gets larger. Its performance begins to be poor when *q*/*ν* is of order 0.1 or higher. In other words, only when the rate of *de novo* emergence of double resistance (in response to combination of both drugs) is one tenth of that of the rate of *de novo* emergence of single resistance (in response to treatment with single drug), does COMBO typically fail in our simulations. While we can not generally exclude the possibility that in some cases *q* is in a similar order of magnitude as *ν*, we do not expect this to be a common scenario [32].

The fitness costs of resistance mutations play a key role in the spread and maintenance of resistance in pathogen populations [48], as confirmed by the magnitude of their effect revealed by the LDA (Fig 4). We thus perform a similar analysis showing how the non-additivity of resistance costs affects the success of combination therapy. Fig 6B shows the fraction of parameter sets for which combination therapy is the best or the worst strategy as a function *c*_3_ − (*c*_1_ + *c*_2_), i.e., the excess cost of double resistance compared to the sum of the costs of single resistance. As can be seen in Fig 6B, as the excess cost of double resistance becomes larger, i.e., as the fitness cost *c*_3_ further exceeds the additive sum of *c*_1_ and *c*_2_, chances for combination therapy to be the best strategy increase as well. If there is no excess cost, i.e., *c*_3_ = *c*_1_ + *c*_2_, COMBO outperforms all other strategies for 62% of the parameter sets. Only when *c*_3_ is much less than the sum of *c*_1_ and *c*_2_, does COMBO fail most of the time. Further analysis on how *q*, *ν*, *c*_1_, *c*_2_, and *c*_3_ change given other treatment strategies win is provided in the supplementary material (S8 Fig).

**Fig 7:**
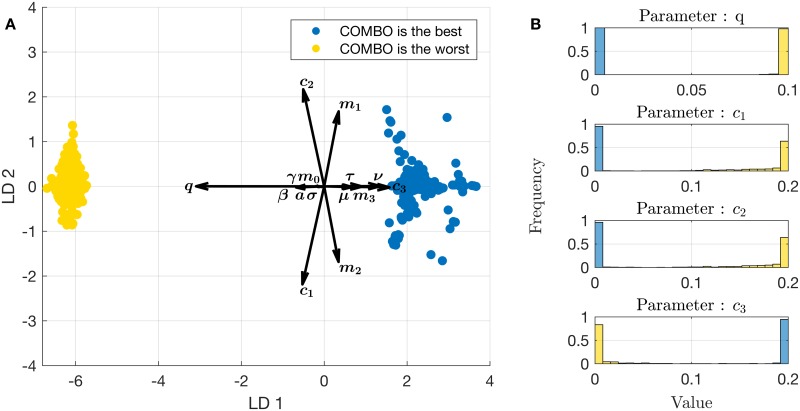
PSO results. PSO results, where 1000 realizations of the PSO algorithm are used for each optimization target. **(A)** PSO results projected on the same LDA space as the RS results. **(B)** Histograms of the most important parameters for each optimization target.

Although random sampling is an efficient way to analyse a large parameter space, it is in general inadequate in detecting any minima or maxima in a complex, non-linear parameter space. To find the parameter sets where combination therapy is the best or the worst strategy, we employ Particle Swarm Optimization (PSO). More precisely, we utilize two different optimization targets to identify two different parameter regions: (i) that for which the efficacy score difference between combination therapy and the second best strategy is maximal (where combination therapy is the best strategy); and (ii) that for which the efficacy score difference between the second worst strategy and combination therapy is maximal (where combination therapy is the worst strategy). These parameter sets are projected onto the LDA space defined by the RS results. The results are shown in Fig 7A. Due to the noisy solution space, different realizations of the PSO algorithm converge to different local extrema, resulting in multiple solutions for both cases. As can be seen from the figure, the points where combination therapy is the worst strategy tend to cluster at the left hand side of the LDA space, in the direction of increasing *q*, and in the opposite direction of the *c*_3_ vector. Similarly, points where combination therapy is the best strategy tend to cluster at the right hand side of the LDA space, showing opposite characteristics in terms of *q* and *c*_3_ values. To have a better understanding of the characteristics of these clusters, we investigate the distributions of the most important parameters *q*, *c*_1_, *c*_2_, and *c*_3_ within each cluster. These distributions are shown in Fig 7B. For both clusters, we observe that the parameters tend to converge to their boundaries given in Table 2, in accordance with what can be predicted from the LDA.

For all simulations used throughout our analysis, simulation duration (*T*) is set to 360 days. However, the time required for all populations to converge to an equilibrium (or oscillate with a certain magnitude in case of CYC) depends on the parameter set that is randomly drawn. Although in most of our simulations populations converge within the course of 360 days, there are also cases where we observe a very slow increase in the number of resistant patients. This raises the question whether the performance of the treatment strategies would alter if the simulation duration is lengthened. Testing this, we find that the relative efficacies of the treatment strategies stay very similar over a broad range of simulation durations. We observe a slight decrease for COMBO (from ∼ 59% for 6 months to ∼ 53% for 120 months) and MIX (from ∼ 24% for 6 months to ∼ 22% for 120 months), and observe a slight increase for MONO-1 and MONO-2 (from ∼ 8% for 6 months to ∼ 11% for 120 months), whereas we observe almost no change for CYC. Therefore we conclude that the simulation duration does not have a significant effect on the choice of optimal treatment strategy.

Two recent theoretical studies have shown that mixing or cycling strategies can be considerably improved if information on the prevalence of resistance is taken into account [34, 41]. While we believe that empiric treatment in the absence of such information remains a clinical reality, one may argue that we are comparing COMBO here with strategies that are known to have considerable potential to be improved when information regarding resistance is taken into account. Following these studies [34, 41] we implemented a further strategy, referred to as REACT, which switches the drug depending on the resistance prevalence in the hospital ward, i.e., assigns drug 1 when *R*_2_ > *R*_1_, and vice versa. Similarly, 500,000 parameter sets are sampled randomly according to the parameter boundaries and sampling schemes given in Table 2. For each parameter set, efficacy scores are calculated for the six treatment strategies, including REACT, and the winning strategy for that particular parameter set is determined. S4 Fig shows the probability that a given strategy is the winning strategy based on all simulation results. As seen from the figure, only the relative efficacy of MIX is highly affected by the introduction of REACT, whereas relative efficacy of COMBO stays almost the same. In agreement with the earlier results [34, 41], our results confirm that an adaptive strategy which takes resistance prevalence into account does perform better than MIX and CYC. On the other hand, COMBO is still the optimal strategy when REACT is introduced. This implies that the strategies under consideration (MIX, CYC, MONO-1, and MONO-2) does not give COMBO an unfair advantage in terms of relative efficacy.

## Discussion

When all strategies including monotherapy are compared, COMBO is the best strategy in 57% of the simulations performed with randomly drawn parameter sets (Fig 3). In the parameter region where it is beneficial to treat a patient with more than one drug, COMBO outperforms CYC and MIX and is the best strategy 70% of the time. Furthermore, by comparing the best with the second best strategy, we find that when COMBO is the best, it tends to outperform the second best strategy by a much larger margin than when another strategy is the best (Fig 5A). However, there are also parameter regions where COMBO is the worst strategy, and in these regions COMBO tends to be considerably worse than the second worst strategy (Fig 5B). We also find that MIX tends to outperform CYC (S2 Fig). We assumed a fixed cycling period of 30 days. The cycling period influences the performance of CYC. For very low cycling periods, CYC behaves like mono-drug therapy, and for very high cycling periods, it converges to MIX [33].

The LDA of simulations with randomly drawn parameters reveals that the rate of *de novo* emergence of double resistance (*q*) and the costs of resistance mutations (*c*_1_, *c*_2_, *c*_3_) are the most important parameters determining which strategy wins (Fig 4). Parameter regions where COMBO fails are characterised by very high values of *q*/*ν* or very low excess cost of double resistance (*c*_3_ − (*c*_1_ + *c*_2_) ≪ 0), which both seem biologically less plausible. In particular, the rate of *de novo* emergence of double resistance, *q*, in response to combination therapy has to be at least one tenth of the rate of *de novo* emergence of single resistance, *ν* (Fig 6A). Similarly, the excess cost of double resistance has to be very small, meaning that the sum of the fitness costs of single resistant strains has to be much higher than the fitness cost of the double resistant strain (Fig 6B). Although *c*_3_ can be decreased to some extent via compensatory mutations [49], the excess cost of double resistance (*c*_3_ − (*c*_1_ + *c*_2_)) must be very small (∼ −0.4) for COMBO to fail.

Our analysis thus suggests that overall COMBO tends to outperform all other strategies in the parameter regions that are considered to be biologically more realistic. Biologically less plausible parameter combinations appear in the analysis since we sample parameter values independently from each other. In reality, we expect many parameters to be coupled. For example, both the recovery rate due to appropriate drug treatment and the rate of *de novo* emergence depend on the drug dose and are hence linked. Moreover, the rate of *de novo* emergence of single and double resistance may in many cases be coupled. While this is a limitation of our approach, it permits us to observe the hypothetical conditions for different outcomes.

LDA has certain caveats. For example, it fails if the discriminatory information is not represented by the mean but the variance of the data. Furthermore, if the distributions are significantly non-Gaussian, projections may not preserve the complex structure in the data needed for classification. However, it is known that LDA frequently performs well even when the assumptions of independence and normality are violated [44]. For our problem, although the assumptions of LDA are not fully satisfied, it is evident from Fig 4 that there is a clear separation of classes.

Since the random sampling of the parameter space could have missed areas where combination therapy fails or succeeds maximally, we used PSO as an optimization strategy to actively search for these particular parameter combinations. The parameter combinations identified by PSO, where COMBO performs best, align well with those found by random sampling (Figs 4 and 7B). Moreover, the regions where COMBO fails or succeeds are at the horizontally opposing ends of the LDA projection. This implies that the random sampling results do not appear to miss relevant parameter regions where COMBO fails or succeeds. Moreover, it highlights that the most relevant parameters separating failure and success are indeed the rate of *de novo* emergence of double resistance (*q*) and the cost of double resistance (*c*_3_). Investigating the PSO results in more detail we see that the important parameters revealed by LDA (*q*, *c*_1_, *c*_2_, and *c*_3_) tend to take their boundary values for COMBO to fail or succeed maximally (Fig 7A). The negative effect of high values of *q* on the relative performance of COMBO was also emphasized previously [32], but was not analysed quantitatively. Although LDA of the random sampling results aligns well with these outcomes, random sampling alone is not necessarily sufficient to determine what parameter values maximize or minimize the success of COMBO. Overall, the congruence of the LDA and the PSO results suggests that the effect of these key parameters are more linear than might have been expected.

Interestingly, we find that the rate of *de novo* emergence of double resistance, *q*, has a strong influence, but the fraction of newly admitted patients carrying double resistance, *m*_3_, has a weak influence on the relative performance of COMBO. The reason for this differential effect is that the process of *de novo* emergence of double resistance only occurs when both drugs are used in combination, whereas the process of admission of double resistance affects all treatment strategies. In other words for large *m*_3_, all strategies fail, whereas for large *q*, only COMBO fails. By allowing *de novo* emergence of double resistance only for COMBO, we bias the model in disfavor of COMBO. In reality, also treatment with a single drug is expected to select for the *de novo* emergence of double resistance.

In order to compare mono- with multi-drug treatment strategies it is necessary to make assumptions about treatment efficacy, drug interactions, side-effects and cross-resistance. In our model we assume, that a strain resistant to drug 1 is equally susceptible to treatment with drug 2 as to treatment with the combination of drug 1 and 2. This is reasonable if we assume (i) that COMBO combines both drugs at the same concentrations as they are used individually and (ii) that there are no drug interactions. We also assume, that there is no benefit of adding a second drug in terms of increasing clearance of the infection. This assumption is conservative by disfavoring COMBO. Finally we assume that the side-effects of all treatment strategies are negligible. While the specific assumptions made are open for discussion, we believe that they are reasonable for a meaningful comparison of mono- versus multi-drug treatment strategies.

By combining earlier models of Bonhoeffer *et al.* (1997) [32] and Bergstrom *et al.* (2004) [33] our model incorporates a broad range of relevant biological aspects and processes such as *de novo* emergence of resistance within the focal population, influx of resistance from outside, single and double resistance, mono- and multi-drug treatment, superinfection and more. Nevertheless, there are further relevant processes that should be considered in future work. For example, on the within-host level, pharmacokinetic and pharmacodynamic effects of the drugs could lead to periods of monotherapy, which are not taken into account in our model. Similarly, penetration profiles of the drugs are not included in the model. Imperfect penetration could increase the chance of treatment failure by creating regions where only one drug from a combination reaches a therapeutic concentration, which results effectively in spatial monotherapy [50]. Also, our model does not consider within-host dynamics of pathogen replication in any greater detail beyond the rates of strain replacement by *de novo* emergence of superinfection. Finally, the model makes simplifying assumptions regarding switching of drugs in treated patients. In more realistic models that specify the rules governing the behaviour of individuals, a patient would remain on the same drug regimen over the entire course of the infection (Uecker and Bonhoeffer, submitted).

Since the eventual goal of treatment is to save as many patient lives as possible, our optimality criterion is based on reducing the overall prevalence of disease. This is a sensible criterion not only with regard to the clinical goals of treatment, but also because it inherently trades off the benefit of using antibiotics with the cost of generating resistance [51]. Optimality criteria that are based solely on the rate of emergence or prevalence of resistance are often used in the literature, but suffer from the fact that the trivial solution—not treating any patient—achieves a maximal score. Our optimality criterion is easily implemented in the context of modeling studies, but the number of infected individuals may be harder to track in a clinical trial. However, we argue that to compare different treatment strategies in a clinical setting, it will also be necessary to use measures that reflect both the positive effects of treatment (e.g. reduced disease prevalence) as well as the negative ones (e.g. increased emergence of resistance).

The analysis of our model over a wide parameter range suggests that with regard to emergence and spread of resistance combination therapy tends to outperform other treatment strategies. Why, then, is the clinical evidence in support of combination therapy not clearer? This may have multiple reasons. First, while our analysis suggests that combination therapy tends to outperform the other strategies, is does not do this for all of the parameter space. We cannot exclude that the relevant parameter space is different from the one studied here, but we have argued above that the parameter region where combination therapy tends to fail seems not to be the biologically most plausible one.

Second, there may be important biological factors missing in our model, which could fundamentally alter the outcome. One such factor may be the acquisition of resistance genes via horizontal gene transfer. A full consideration of horizontal gene transfer would require the inclusion of explicit equations for plasmids as well as the commensal bacterial population. The probability of acquiring double compared to single resistance via horizontal gene transfer is likely not the product of the individual probabilities of single resistance as would be expected for resistance acquired through spontaneous chromosomal mutations. This is in part the reason why we chose to have independent rates of *de novo* resistance of single and double resistance in our model. However, there may be yet other factors that could be relevant, and identifying biological factors that would fundamentally change the picture regarding optimal treatment strategies would represent an important contribution to the field.

Finally, the discrepancy between the model favoring combination therapy and clinical practice by enlarge favoring monotherapies, may also be due to the lack of appropriate data. Most clinical studies comparing combination therapy to monotherapy use mortality as clinical endpoint and show either some advantage or non-inferiority [13–21] (see also Introduction). However, the effect of these therapies on the emergence of resistance is by nature a much more long term outcome, considerably harder to assess, and the evidence both for or against combination therapy thus comparably weak. Mathematical models are thus an important tool to address this knowledge gap and suggest that combination therapy deserves serious consideration as a strategy to delay the emergence of resistance.

## Acknowledgement

We gratefully acknowledge Balázs Bogos for valuable comments and discussions.

## Supporting information

S1 Table Statistics on efficacy scores for multi-drug therapies. Probability of being the worst and the best strategy is provided with the minimum, maximum, and mean efficacy score for each multi-drug therapy. For the chosen parameter range COMBO has a lower average score than MIX or CYC. Moreover, it has not only the highest probability to be the best strategy, but also the highest probability to be the worst strategy. Note, however, that COMBO tends to outperform the other strategies in regions that are considered to be more realistic and tends to lose in regions that are considered less realistic (see S1 Fig; see also the Discussion section in the main text and Fig 5).
(EPS)

S1 Fig Correlation of efficacy scores. Correlation of efficacy scores for different pairs of strategies, where all (500,000) random sampling results are used. The correlation coefficient is given in the upper left corner of each panel. Points below and above *x* = *y* line are colored differently, and the number of points of each color is given in the lower right legend. Correlation of efficacy scores: **(A)** Best against worst strategy, **(B)** CYC against MIX, **(C)** CYC against COMBO, **(D)** MIX against COMBO. The high correlation between efficacy scores implies that choice of parameters has a much stronger influence on determining scores than the chosen treatment strategy. Example simulations for small and large differences between the efficacy scores of the treatment strategies are found in S3 Fig.
(EPS)

S2 Fig Marginal benefit comparisons for different pairs of multi-drug strategies. Disks are colored according to the better strategy, and both the disk size and the transparency are proportional to the efficacy score difference between the strategies in comparison. LD1 and LD2 are the two principal axes of the LDA. 2000 random samples are used for each panel. **(A)** Comparison between CYC and COMBO. **(B)** Comparison between CYC and MIX. **(C)** Comparison between MIX and COMBO.
(EPS)

S3 Fig Time behaviour of the patient populations for a given parameter set, under MIX, CYC, COMBO, MONO-1 and MONO-2. Applied therapy and its efficacy score is given in the title of each subfigure. **(A)** The parameter set is chosen such that there is a negligible difference between the efficacy scores of strategies, aiming to demonstrate a case where there is no clear winning or losing strategy. **(B)** The parameter set is chosen such that there is a substantial difference between the efficacy scores of strategies, aiming to demonstrate a case where there is a clear winning strategy.
(EPS)

S4 Fig Chance of being the best strategy, where additionally reactive cycling (REACT) is implemented. 500,000 randomly sampled parameter sets are used. For each parameter set, efficacy scores are calculated for the six treatment strategies, and the winning strategy for that particular parameter set is determined. Based on these results, the probability of being the best strategy for each treatment is calculated.
(EPS)

S5 Fig Histograms of the fraction of different patient groups at the treatment-free steady state. Histogram of the fraction of different patient groups at the treatment-free steady state given **(A)** MIX, **(B)** CYC, **(C)** COMBO, **(D)** MONO-1, and **(E)** MONO-2 wins. **(F)** Unconditional histogram of the fraction of different patient groups at the treatment-free steady state.
(EPS)

S6 FigChance of being the best strategy according to the random sampling results for randomly drawn initial conditions.500,000 randomly sampled parameter sets are used, and the initial conditions for the fraction different patient groups (*S*(0), *R*_1_(0), *R*_2_(0), *R*_3_(0), and *X*(0)) are randomly drawn from U(0,1) under the constraint *S*(0) + *R*_1_(0) + *R*_2_(0) + *R*_3_(0) + *X*(0) = 1. For each parameter set, efficacy scores are calculated for the five treatment strategies, and the winning strategy for that particular parameter set is determined. Based on these results, the probability of being the best strategy for each treatment is calculated.(EPS)

S7 Fig LDA of random sampling results for multi-drug therapies. LDA classifying parameter sets according to the strategy with the highest efficacy score, when efficacy scores are calculated only for MIX, CYC, and COMBO. Shaded areas represent the density of the parameter sets colored according to which strategy wins. Each treatment strategy is represented by a different color, and the opacity of each color is proportional to the number of parameter sets that fall into that corresponding region. 500,000 randomly sampled parameter sets are used. LD1 and LD2 are the two principal axes of the LDA. The parameter vectors are given with their relative magnitudes and counterclockwise angles. Parameter vectors are amplified by a factor of 3 in order to make them better visible.
(EPS)

S8 Fig Histograms of the important parameters given a treatment strategy wins. 500,000 randomly sampled parameter sets are used, and histograms for the important parameters *q*, *ν*, *c*_1_, *c*_2_, and *c*_3_ are calculated given a certain treatment strategy wins. The lines in each panel are colored according to the winning strategy. For each parameter, 10 equally spaced values between its corresponding boundaries are used for binning. Histograms are shown as lines for better visibility. Histogram of **(A)** log(*q*), **(B)** log(*ν*), **(C)** *c*_1_, **(D)** *c*_2_, and **(E)** *c*_3_, given a certain treatment strategy wins.
(EPS)
